# Genetically-Defined Deficiency of Mannose-Binding Lectin Is Associated with Protection after Experimental Stroke in Mice and Outcome in Human Stroke

**DOI:** 10.1371/journal.pone.0008433

**Published:** 2010-02-03

**Authors:** Alvaro Cervera, Anna M. Planas, Carles Justicia, Xabier Urra, Jens C. Jensenius, Ferran Torres, Francisco Lozano, Angel Chamorro

**Affiliations:** 1 Comprehensive Stroke Center, Hospital Clínic, Institut d'Investigacions Biomèdiques August Pi i Sunyer (IDIBAPS), Medical School, University of Barcelona, Barcelona, Spain; 2 Department of Brain Ischemia and Neurodegeneration, Institut d'Investigacions Biomèdiques de Barcelona (IIBB)-Consejo Superior de Investigaciones Científicas (CSIC), Institut d'Investigacions Biomèdiques August Pi i Sunyer (IDIBAPS), Barcelona, Spain; 3 Department of Medical Microbiology and Immunology, University of Aarhus, Aarhus, Denmark; 4 Clinical Pharmacology Unit, Hospital Clínic, Institut d'Investigacions Biomèdiques August Pi i Sunyer (IDIBAPS), Medical School, University of Barcelona, Barcelona, Spain; 5 Department of Cellular Biology, Immunology, and Neuroscience, Medical School, University of Barcelona, Barcelona, Spain; 6 Immunology Department, Hospital Clínic, Institut d'Investigacions Biomèdiques August Pi i Sunyer (IDIBAPS), Barcelona, Spain; Charité Universitaetsmedizin Berlin, Germany

## Abstract

**Background:**

The complement system is a major effector of innate immunity that has been involved in stroke brain damage. Complement activation occurs through the classical, alternative and lectin pathways. The latter is initiated by mannose-binding lectin (MBL) and MBL-associated serine proteases (MASPs). Here we investigated whether the lectin pathway contributes to stroke outcome in mice and humans.

**Methodology/Principal Findings:**

Focal cerebral ischemia/reperfusion in MBL-null mice induced smaller infarctions, better functional outcome, and diminished C3 deposition and neutrophil infiltration than in wild-type mice. Accordingly, reconstitution of MBL-null mice with recombinant human MBL (rhMBL) enhanced brain damage. In order to investigate the clinical relevance of these experimental observations, a study of MBL2 and MASP-2 gene polymorphism rendering the lectin pathway dysfunctional was performed in 135 stroke patients. In logistic regression adjusted for age, gender and initial stroke severity, unfavourable outcome at 3 months was associated with MBL-sufficient genotype (OR 10.85, p = 0.008) and circulating MBL levels (OR 1.29, p = 0.04). Individuals carrying MBL-low genotypes (17.8%) had lower C3, C4, and CRP levels, and the proinflammatory cytokine profile was attenuated versus MBL-sufficient genotypes.

**Conclusions/Significance:**

In conclusion, genetically defined MBL-deficiency is associated with a better outcome after acute stroke in mice and humans.

## Introduction

Innate immunity presents the first line of defense against infections through the recognition of pathogen-associated molecular patterns. Innate immunity also recognizes molecular motifs expressed by stressed or dead host cells following a general system of response referred to as innate autoimmunity [1]. The complement system is one of the major effector mechanisms of the innate immunity and its activation contributes to the release of inflammatory mediators, opsonophagocytosis and killing of pathogens and removal of damaged host cells. The complement system is activated after acute brain injury, and astrocytes, microglia, neurons, and oligodendrocytes are able to synthesize several complement components [2]. Recently, some clinical studies have provided evidence supporting activation of the complement system in patients with acute stroke [3], [4].

Crucial steps of the innate response entail the activation of the alternative pathway and the lectin pathway of the complement system, to converge in the generation of enzyme complexes that are able to cleave C3, which is the most abundant complement protein [5]. In experimental stroke, the complement system is also activated through non classical pathways (either the alternative or the lectin pathways) [6], [7]. Complement activation appears to contribute to the development of larger brain infarctions [8], hence depletion of complement results in beneficial effects [9], [10], more readily observed in situations where the brain ischemia is followed by reperfusion [11]. However, the mechanism and the clinical relevance of complement activation in human stroke remains unsettled.

Mannose-binding lectin (MBL) is a C-type collectin homologous to C1q that can initiate the activation of complement through the lectin pathway. This is an ancient and highly conserved pathway of complement activation that has been implicated in the pathophysiology of myocardial infarction [12], gastrointestinal ischemia [13], and kidney ischemia/reperfusion [14]. A recent study shows the benefits of C1 inhibitor administration in a murine model of cerebral ischemia/reperfusion and suggests that MBL is involved in this effect [15]. The activation of the lectin pathway is mediated by associated serine proteases, termed MASPs, since they were discovered as MBL-associated serine proteases [16]. Of those, MASP-2 is essential as it cleaves C4 and C2 components, leading to the formation of the classical C3 convertase [17]. MBL can also recognize and bind damaged host cells [18] and it is possible that this collectin might also recognize damaged cells in the central nervous system.

Low serum levels of MBL are reported in one in five persons of European descent due to the presence of single nucleotide polymorphisms (SNPs) in the promoter and the structural coding region of the *MBL2* gene [17], [19]. A SNP in the *MASP2* gene (D105>G) may also result in low circulating levels of MASP-2 [20] but little is known of its pathogenic consequences after experimental or clinical stroke. The worldwide expansion of a few *MBL2* mutant alleles that lead to low serum levels of MBL has led to the suggestion that they might be advantageous under some circumstances [21]. In this study, we investigated the clinical implications of genetic polymorphisms of key components of the lectin pathway (*MBL2/MASP2*) in patients with acute stroke. We also assessed the consequences of acute ischemia in MBL-null mice and wild-type mice. To our knowledge, the results of this study underscore for the first time the central role of the lectin pathway of complement activation after acute stroke in clinical and experimental settings and illustrate that a richer expression of this soluble pattern recognition molecule in the innate immune system facilitates a stronger inflammatory response after acute brain injury.

## Materials and Methods

### Brain Ischemia in Mice

Four-month-old male MBL-null mice (B6.129S4-Mbl1tm1Kata Mbl2tm1Kata/J) were obtained from The Jackson's Laboratory. Age-matched male C57BL/6J wild-type (WT) mice were used as controls. Animal work was approved by the local Ethical Committee (Comité Ético de Experimentación Animal, University of Barcelona). Mice were anesthetized with isoflurane and brain ischemia was induced by 2-hour occlusion of the middle cerebral artery (MCA), as reported [22]. Cortical perfusion was evaluated with laser Doppler flowmetry (Perimed, Sweden). Neurological deficits were assessed at 48 h. Infarct volume was measured at 48 h in 1 mm-thick coronal brain sections stained with 1% solution of 2,3,5- triphenyltetrazolium chloride (TTC). The area of infarction [23] (pale zone not turning red after staining) was measured in each section and a correction for oedema was made by multiplying the infarct area by the ratio of the contralateral to the ipsilateral hemisphere. The areas were integrated to calculate infarct volume. In each brain section, we calculated the percentage of hemispheric area that was infarcted by dividing the infarct area by the area of the contralateral hemisphere *100 (See Text S1).

### Reconstitution with rhMBL

An additional group of MBL-null mice received either endotoxin-free rhMBL or vehicle (tris-buffered saline, TBS). The rhMBL was generously supplied by NatImmune A/S, Copenhagen. It was produced by transfected HEK293F cells and purified by carbohydrate affinity chromatography and gel permeation chromatography. DNA was removed by Benzonase treatment; and several microfiltration and nanofiltration steps were done to reduce bioburden and elimination of adventitious virus. No sign of side effects such as would be expected from endotoxin contamination were seen in pre-clinical or phase I trials [24]. rhMBL (200 µg in 200 µl of TBS) or vehicle was given i.p. 4 times, i.e. 24 h, 14 h, and 2 h prior to MCA occlusion and 12 h later [25]. Researchers inducing ischemia and measuring infarct volume were not aware of the treatment assigned to each animal.

### Immunohistochemistry and Western Blotting

Immunohistochemistry was performed in cryostat sections obtained at 48 h. A rabbit polyclonal antibody against C3 (Abcam, Cambridge, UK) was used diluted 1∶500. The percent immunopositive area for each animal was measured using AnalySIS Software (Soft Imaging System). Quantification of C3 deposition was made in sections taken at three different coronal sections form Bregma +2 mm to Bregma −3 mm. Images were taken from 3 fields and the mean value was calculated for each animal. The area of each field was 0.32 mm^2^. Images were taken from the zones showing the highest intensity of fluorescence per each section. The sections were labeled with a code that did not reveal the identity of the animals. Brain protein extracts were obtained from frozen brain tissue and processed for Western blotting [22]. Two different rabbit anti-mouse antibodies against C3 (from Abcam and from Santa Cruz Biotechnology Inc., CA, USA) were used. Further details are given in Text S1.

### Stroke Patients

Patients were part of the ESPIAS stroke trial [26]. Subjects gave specific written consent for genetic analyses. The study was conducted according to the Declaration of Helsinki principles and approved by an official Internal Review Board (Ethic Committee of Clinical Investigation of the Hospital Clinic of Barcelona; number of approval: HCP 01/01237). A modified Rankin Scale (MRS)<2, NIHSS score<2, and Barthel Index of 95 or 100 at 3 months indicated a favourable outcome. Another outcome measure was the incidence of infections during the first week [26].

Blood was collected at baseline (day 0) and days 1, 2, 3, 4, 7, and 90, and serum was stored at −80°C. The concentration of leukocytes, C3 and C4, and of several cytokines and other molecules was quantified (see Text S1). The balance between T helper 1 (Th1) and Th2 cytokines was assessed by the relationship between circulating cytokine levels according to the calculation (TNF-α+IL-6)/IL-10.

### Genetic and Functional Assessment of the Lectin Pathway

At day 0, genomic DNA was extracted. *MBL2 and MASP2* were genotyped with reported sequence-based typing technique [27] (see Text S1). According to previous studies [19], [28], [29], genotypes 0/0, 0/XA, and XA/XA were classified as MBL-low variants while the remainders were MBL-sufficient variants. For *MASP2 gene*, SNP at codon 105 exon 3 implies a non-synonymous amino acid replacement (D105>G) at the CUB1 domain of MASP-2, which impedes association to MBL and significantly reduces MASP-2 levels.

Serum concentrations (ng/ml) of MBL and MASP-2 were determined by time-resolved immunofluorometric assay (TRIFMA) [30], [31] (see Text S1).

### Statistical Analyses

Categorical data were compared using the χ^2^ and Fisher's exact tests. Continuous variables were analyzed with the Student's t-test, non-parametric Mann-Whitney U-test, and Pearson or Spearman coefficients when required. ANCOVA adjusted for baseline values was used to assess serial changes of inflammatory parameters according to genotype. Two-way ANOVA was applied for comparisons of the percentage of infarcted area between MBL-null mice and WT mice for the different brain sections. Changes over time of MBL and MASP-2 levels were analysed with a longitudinal mixed model for repeated measurements that included genotype, time and their interaction in the model, and setting the type of covariance as unstructured. Logistic regression modeling was used to assess baseline predictors of functional independence at day 90 adjusted for the effects of age, gender and baseline NIHSS score.

## Results

### MBL-Deficiency Is Beneficial in Experimental Brain Ischemia

**Figure 1:**
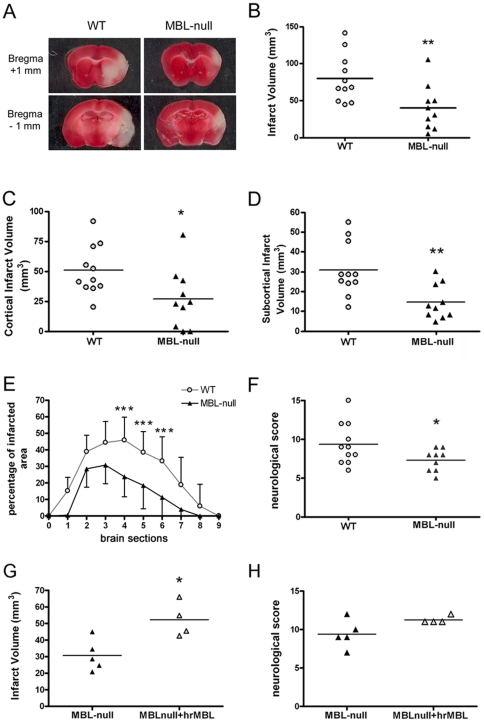
MBL-null mice develop smaller infarct volumes than wild-type mice. A) Representative images of infarcted brain tissue in wild-type (WT) and MBL-null mice at 48 h post-ischemia. The approximate level of the brain sections is indicated in the left by the distance from Bregma. B) Infarct volume was lesser in the MBL-null group (n = 11) than in WT (n = 10). C) Cortical and D) subcortical infarct volumes were smaller in MBL-null than in WT mice. E) The percentage of tissue with infarction per brain section (1 mm-thick) is shown for consecutive sections, starting (left) from the frontal part of the brain. The proportion of infarcted tissue is smaller in MBL-null than in WT mice. Values are expressed as the mean±SD. Two-way ANOVA by genotype and brain section showed significant differences due to genotype (p<0.001, F(9,40) = 18.74). Post-hoc Bonferroni test showed significant differences in the indicated brain sections. F) The neurological score was better (lower) in MBL-null mice than in WT. (G–H) MBL-null mice were treated with rhMBL (MBLnull+rhMBL) (n = 4) or vehicle (MBLnull) (n = 5). G) MBL-null mice receiving rhMBL showed larger infarct volume than non-reconstituted mice. H) A trend to worst neurological deficit (p = 0.09) was seen after reconstitution with rhMBL compared to vehicle. Symbols indicate values for individual animals. ^*^ p<0.05, ** p<0.01, *** p<0.001).

Brain damage after 2-hour middle cerebral artery occlusion (MCA) was assessed by measuring infarct volume at 48 h in WT (n = 10) and MBL-null (n = 11) mice. Representative images of infarction in WT and MBL-null mice are shown in Fig. 1A. Infarct volume was significantly reduced (p<0.05) in the MBL-null group (Fig. 1B). The effects were significant in cortical (p<0.05) (Fig. 1C) and subcortical (p<0.01) (Fig. 1D) structures. However, as the mean±SD body weight of MBL-null mice (26.50±0.46 g) was smaller than that of WT mice (29.05±0.93 g) (p<0.001), we made some calculations to reassure that the reduction in body size in the MBL-null mice did not contribute to the differences in infarct volume vs. WT. In each brain section, we calculated the percentage of hemispheric area that was infarcted by dividing the infarct area by the area of the contralateral hemisphere *100. The results (Fig. 1E) showed that the proportion of brain tissue that was infarcted in the different brain sections was smaller in MBL-null mice than in WT (p<0.001, two-way ANOVA by genotype and brain section). In agreement with the reduction in infarct size, the neurological test studied at 48 h showed that the functional deficit was minor in MBL-null mice than in WT mice (p = 0.05) (Fig. 1F).

The magnitude of the ischemic lesion is dependent on the reduction of cerebral blood flow (CBF) experimentally induced by occlusion of the middle cerebral artery (MCA). For this reason, we verified that MBL-deficient mice responded to MCA occlusion with a similar perfusion deficit than WT mice by monitoring CBF with laser Doppler flowmetry. The results showed that CBF (mean±SD) dropped until 30±8% and 32±7% of baseline in WT mice (n = 6) and in MBL-null mice (n = 8), respectively. The results illustrated that MBL-deficiency did not affect (p = 0.61) the CBF response to MCA occlusion and, thus, that the severity of the induced ischemia was similar in WT and MBL-null mice.

We then carried reconstitution experiments by administering human recombinant MBL (rhMBL) protein to MBL-null mice to validate that the smaller infarct volume in these animals was attributable to the lack of MBL. rhMBL or vehicle was administered i.p. following a reconstitution protocol that rescues MBL pathway activity in plasma [25]. MBL-null mice that were reconstituted with rhMBL showed a significantly larger infarction than mice of the vehicle group (p<0.05) (Fig. 1G), in spite that a similar CBF reduction after ischemia was observed after rhMBL or vehicle (p = 0.84). Also, a trend was observed for a worst neurological score in the rhMBL group, but the difference compared with vehicle did not reach statistic significance (p = 0.09) (Fig. 1H).

### Post-Ischemic Complement Activation Was Attenuated in MBL-Null Mice vs. WT

**Figure 2:**
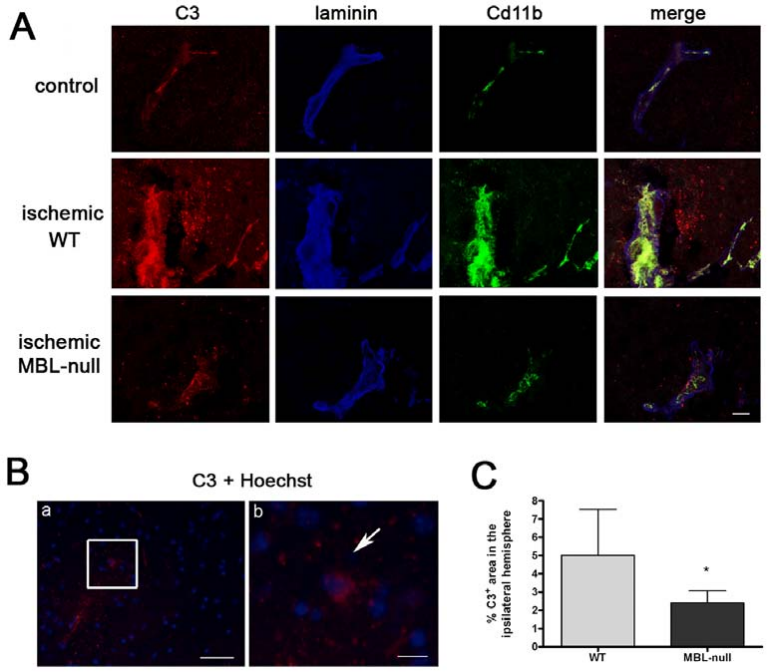
Ischemia-induced C3 deposition in brain parenchyma is attenuated in MBL-null versus WT mice. A) C3 deposition (red), laminin immunostaining (blue), and CD11b (green). C3 immunoreaction is often associated to vessels and it is also found within the ischemic brain parenchyma. The C3 reaction is more moderate in MBL-null mice than in the WT. Control indicates non-operated WT mice. Bar scale  = 10 µm. B) C3 deposition (red) and Hoechst counterstaining to illustrate the cell nuclei shows C3 immunoreaction surrounding certain cell bodies (arrow in b). (b) is a magnification of the square shown in (a). C) Quantification of the C3 immunoreactive area in the ipsilateral hemisphere shows a significant reduction in MBL-null mice (n = 5) versus the WT (n = 5). Values are expressed as the mean±SD. Bar scale: (a) 100 µm, (b) 10 µm. * indicates p<0.05.

We examined the deposition of C3 complement protein in mouse brain by means of immunohistochemistry in frozen sections of brain tissue obtained at 48 h post-ischemia. C3 immunoreactivity was observed in blood vessels and also within the brain parenchyma in the ipsilateral hemisphere of WT mice, and, to a lesser extent, of MBL-null mice (Fig. 2A). Counterstaining the C3 immunoreaction with Hoechst showed C3 positive reaction surrounding certain cell bodies (Fig. 2B). For a quantitative assessment of differences in C3 immunoreactivity between groups we measured the C3-positive area in microscope images taken from the ischemic zone. The results (Fig. 2C) showed that the C3-immunoreactive area was smaller in MBL-null (n = 5) than in WT (n = 5) mice, thus supporting that the extent of complement activation was milder in MBL-null than in WT mice.

**Figure 3:**
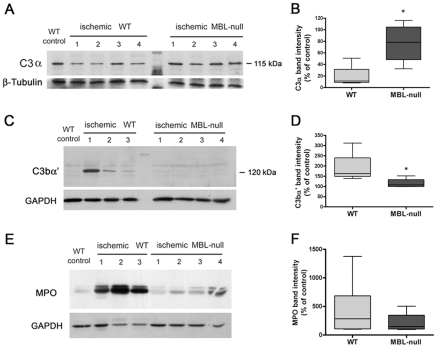
C3 activation and neutrophil infiltration in the ischemic brain is lower in MBL-null than WT mice. A) C3 α-chain is observed in control tissue with a rabbit anti-mouse C3 antibody. C3 is cleaved after ischemia in WT mice and, to a lesser extent, in MBL-null mice. Lanes represent different animals. B) Quantification of band optical density shows more severe cleavage of the C3 α-chain in WT than in MBL-null mice. C) The C3bα' fragment (120 kDa) is evidenced in the ischemic tissue with an anti-C3 antibody. D) Quantification of band optical density shows that C3bα' is more abundant in the ischemic tissue of WT than of MBL-null mice. E) Myeloperoxidase (MPO) is a marker of neutrophil infiltration in brain tissue. MPO is detected in the ischemic brain of WT mice, while it is attenuated in MBL-KO mice, as shown in F). Values are expressed as mean±SD. * p<0.05.

Native C3 is a heterodimeric protein made of one α-chain (115-kDa) and one β-chain. C3 activation causes cleavage of C3α and generation of an active C3a fragment (9 kDa) and the remaining fragment named C3bα'. The latter can covalently bind to small target proteins and acquire a greater apparent molecular mass. Evidence for C3 cleavage after brain ischemia was obtained by studying frozen brain tissue that was homogenized with detergent and examined by Western blotting using development with anti-C3 antibody. In the ischemic brain tissue of WT mice, the full length C3 protein and the C3α chain were degraded, as shown in Fig. 3A, suggesting cleavage of the protein. This effect occurred to a lesser extent in ischemic MBL-null mice (Fig. 3A and B). Moreover, we detected a C3 fragment of around 120 kDa in the ischemic brain tissue of WT mice, which was absent in the control brain, and less abundant in MBL-null than in WT mice (Fig. 3C and D). This protein had a molecular weight compatible with C3bα'.

As an indicator of neutrophil accumulation in the ischemic brain, expression of myeloperoxidase (MPO) was assessed by Western blotting in frozen brain tissue using an anti-MPO antibody. Ischemia induced MPO accumulation in the brain of WT mice, and this effect was milder in MBL-null mice (Fig. 3E and F), suggesting that neutrophil accumulation after ischemia was attenuated in the brain of MBL-deficient animals.

### The Mannose-Pathway in Human Stroke

We undertook investigations to find out whether the neuroprotective effects of MBL-deficiency found in experimental brain ischemia are relevant to human stroke. For this purpose, we studied the genotype, and measured MBL levels, together with C3 and C4 complement proteins, and several markers of inflammation in the serum of our patients. One-hundred and thirty five patients completed the study, including 109 (80.7%) ischemic strokes and 26 (19.3%) hemorrhagic strokes. Patients with ischemic or hemorrhagic stroke disclosed no significant differences in demographics, risk factors, stroke severity on admission, clinical course, MBL and MAPS2 genotype and levels (see Table S1). Infections during the first 7 days after stroke were observed in 24 (17.8%) patients, functional independence at day 90 was reached by 47 (34.8%) patients, and there were 25 deaths at day 90 (19%). Other general traits of these patients were as previously reported [26].

**Figure 4:**
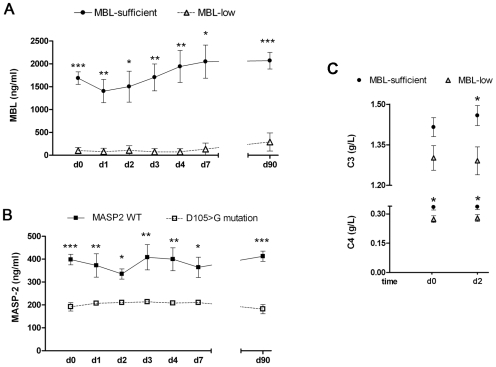
Serum levels of MBL and MASP-2 and complement system activation according to *MBL2* and *MASP2* genotypes. A) Serum concentrations (ng/ml) of MBL in MBL-low genotypes and MBL-sufficient genotypes. B) Serum concentrations (ng/ml) of MASP-2 in patients with D105>G or wild-type *MASP2* genotypes. Measurements were performed at day 0 (d0) (n = 96), day 1 (d1) (n = 10), day 2 (d2) (n = 7), day 3 (d3) (n = 10), day 4 (d4) (n = 10), day 7 (d7) (n = 9) and day 90 (d90) (n = 96). C) Serum concentration (g/L) of C3 and C4 at day 0 (d0) (n = 96) and day 2 (d2) (n = 96). Values are the mean ±SD. *p<0.05; **p<0.01; ***p<0.0001.

In our study, twenty-four (17.8%) patients had genetic MBL-low variants. These patients showed lower serum levels of MBL protein than the group with genetic MBL-sufficient variants (Fig. 4A). MBL levels in serum disclosed no major fluctuations over time after stroke onset (p = 0.79). In addition, fifteen (11.1%) patients were heterozygous for the D105>G SNP at the *MASP2* gene, while none of the patients were homozygous. This SNP has been linked to a severe immunodeficiency when presented in homozygousity [20], [32]. Patients with this *MASP2* polymorphism disclosed lower levels of MASP-2 protein (Fig. 4B). Overall, there were no significant changes in circulating levels of MASP-2 over time (p = 0.82). Demographics, risk factors, clinical parameters at admission, and treatment allocation were evenly distributed according to *MBL2* or *MASP2* genotypes, as shown in Table 1.

**Table 1:** Baseline characteristics in the study population (n = 135) according to MBL and MASP2 genotype.

	MBL-low	MBL-sufficient	p value	D105>G	MASP2-WT	p value
	n (%)	n (%)		n (%)	n (%)	
	24 (18)	111 (82)		15 (11)	120 (89)	
Age (mean, SD), yrs	74 (13)	73 (12)	0.67	70 (15)	73 (11)	0.48
Male, no.(%)	9 (38)	59 (53)	0.16	7 (47)	61 (51)	0.49
Active smoking, no. (%)	3 (13)	20 (18)	0.77	3 (20)	20 (17)	0.39
Hypertension, no. (%)	12 (50)	73 (66)	0.15	6 (40)	79 (66)	0.06
Diabetes, no. (%)	6 (25)	24 (22)	0.72	5 (33)	25 (21)	0.27
Coronary heart disease, no. (%)	2 (8)	15 (14)	0.49	1 (7)	16 (13)	0.22
Previous stroke, no. (%)	4 (17)	19 (17)	1.00	0 (0)	23 (19)	0.06
Peripheral artery disease, no. (%)	2 (8)	9 (8)	1.00	0 (0)	11 (9)	0.22
Admission NIHSS score, mean. (SD)	12 (7)	14 (7)	0.10	14 (7)	13 (7)	0.60
Hemorrhagic stroke at onset, no. (%)	6 (25)	20 (18)	0.40	3 (20)	23 (19)	0.93
Systolic BP (mean, SD), mm Hg	165 (39)	163 (30)	0.80	160 (34)	164 (32)	0.69
Diastolic BP (mean, SD), mm Hg	87 (21)	89 (20)	0.71	94 (24)	88 (19)	0.52
Glucose (mean, SD), mg/dL	141 (36)	145 (54)	0.69	166 (70)	141 (47)	0.19
Axillary temperature (mean, SD), °C	36 (0.5)	36 (0.5)	0.32	36 (0.5)	36 (0.5)	0.35
Treatment with levofloxacin, no. (%)	12 (50)	55 (50)	0.97	10 (67)	57 (48)	0.16

Patients with MBL-low genotypes disclosed lower serum levels of C3 and C4 than patients with MBL-sufficient genotypes (Fig. 4C). Likewise, MBL levels were positively correlated with C3 at different time points after stroke onset, i.e. day 0 (r = 0.23, p = 0.01) and at day 2 (r = 0.34, p<0.01), and C4 at day 0 (r = 0.31, p<0.01), and at day 2 (r = 0.36, p = 0.001). In contrast, the *MASP2* genotype did not influence the levels of C3 and C4 after stroke (Fig. S1).

### Circulating C-Reactive Protein and Cytokine Profile of Stroke Patients According to Their Genotype

**Figure 5:**
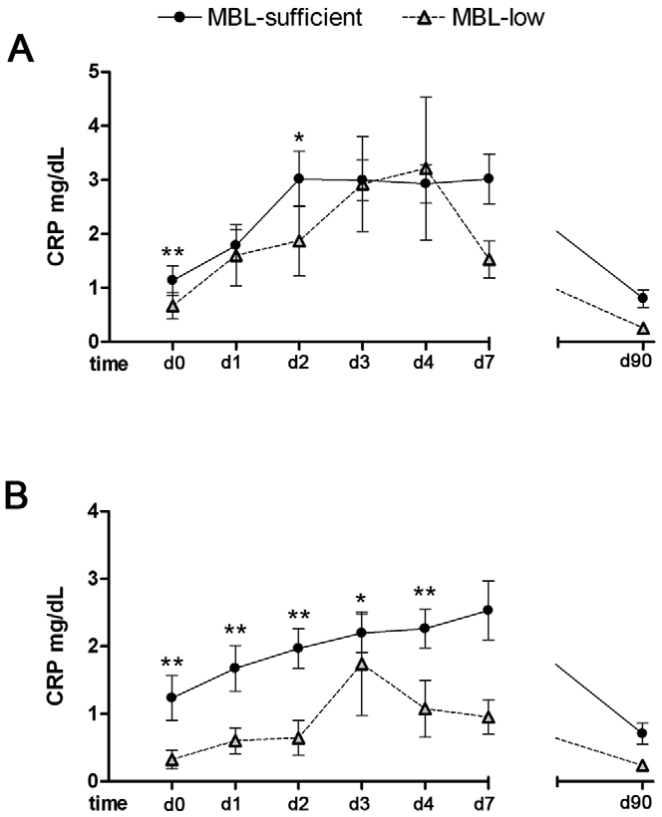
C-reactive protein levels (mg/dL) in MBL-low genotypes and MBL-sufficient genotypes. A) All patients, day 0 (d0) baseline (n = 127; MBL-low = 23, MBL-sufficient = 104); day 1 (d1) (n = 131; MBL-low = 24, MBL-sufficient = 107); day 2 (d2) (n = 132; MBL-low = 24, MBL-sufficient = 108); day 3 (d3) (n = 127; MBL-low = 24, MBL-sufficient = 103); day 4 (d4) (n = 122; MBL-low = 22, MBL-sufficient = 100), day 7 (d7) (n = 111; MBL-low = 19, MBL-sufficient = 92), and day 90 (d90) (n = 68; MBL-low = 12, MBL-sufficient = 56). B) Patients without post-stroke infections, d0 (n = 103; MBL-low = 18, MBL-sufficient = 85); d1 (n = 107; MBL-low = 19, MBL-sufficient = 88); d2 (n = 108; MBL-low = 19, MBL-sufficient = 89); d3 (n = 105; MBL-low = 19, MBL-sufficient = 86); d4 (n = 101; MBL-low = 17, MBL-sufficient = 84), d7 (n = 91; MBL-low = 15, MBL-sufficient = 76), and d90 (n = 60; MBL-low = 11, MBL-sufficient = 49). Values were obtained from serum and are expressed as the mean ± SD. * p<0.05, ** p = 0.01. *p<0.05, **p = 0.01.

CRP levels were lower in patients with MBL-low genotypes compared to MBL-sufficient genotype (Fig. 5A), but were not related with the *MASP2* genotype (data not shown). In exploratory analyses restricted to patients without infections, a lower rise of CRP was confirmed in patients with MBL-low variants (Fig. 5B). Expectedly, CRP and IL-6 were highly correlated (r = 0.37, p<0.0001). CRP was also positively correlated with C3 (r = 0.44, p<0.0001), and C4 (r = 0.33, p<0.01).

**Figure 6:**
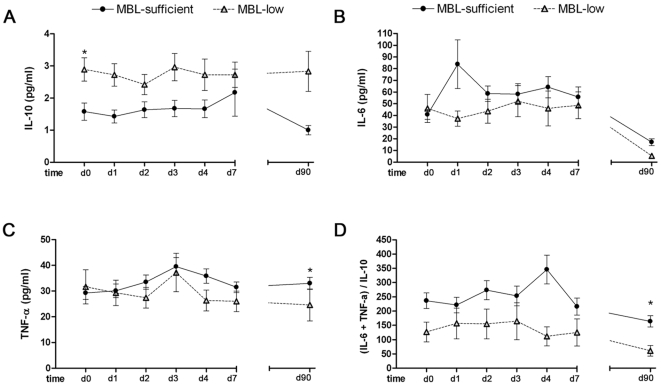
Cytokine levels (pg/ml) in blood in MBL-low genotypes and MBL-sufficient genotypes. A) Interleukin-10; B) Interleukin-6; C) TNF-α; D) Balance between T helper (h) 1 cytokines and Th2 cytokines calculated as (TNF-α + IL-6)/IL-10. Measurements were performed in serum samples at day 0 (d0) (n = 129; MBL-low = 23, MBL-sufficient = 106); day1 (d1) (n = 121; MBL-low = 22, MBL-sufficient = 99); day 2 (d2) (n = 122; MBL-low = 22, MBL-sufficient = 100); day 3 (d3) (n = 116; MBL-low = 19, MBL-sufficient = 97); day 4 (d4) (n = 111; MBL-low = 16, MBL-sufficient = 95), day 7 (d7) (n = 101; MBL-low = 16, MBL-sufficient = 85), and day 90 (d90) (n = 92; MBL-low = 15, MBL-sufficient = 77). Values are the mean ± SD. *p<0.05, **p<0.01.

MBL-low patients showed a cytokine profile after stroke characterized by a greater increase of IL-10, a lower rise of IL-6, and no significant changes of TNF-α compared to patients with MBL-sufficient genotypes (Fig. 6A–D). This predominant anti-inflammatory cytokine response remained significant 3 months after stroke onset. The temporal course of other immune players, such as neutrophils, monocytes, and lymphocytes, did not show a significant effect related to *MASP2* or *MBL2* genotypes (data not shown).

### Stroke Recovery in Patients

Functional independence at day 90 was observed in 14 patients (58.3%) with MBL-low, and in 33 patients (29.7%) with MBL-sufficient genotype (χ^2^, p = 0.008). Unfavourable outcome at 3 months after stroke was associated with MBL-sufficient genotype (OR 10.85, 95% CI 62.94–1.87, p = 0.008) and MBL levels (OR 1.29, 95% CI 1.69–1.02, for every 500 units increase, p = 0.04) in a logistic regression model adjusted for age (OR 1.06, 95% CI 1.00–1.12, p = 0.04), sex (OR 0.32, 95% CI 0.09–1.16, versus male gender, p = 0.08), and initial stroke severity (OR 4.14, 95% CI 2.20–7.88, for each quartile of NIHSS score, p<0.0001). The MASP2-sufficient genotype (OR 0.92, 95% CI 0.23–3.73, p = 0.91) was not associated with stroke recovery. And last, patients with unfavourable outcome showed a predominantly pro-inflammatory cytokine profile (Fig. S2).

Post-stroke infections at day 7 were observed in 5 (20.8%) patients with MBL-low genotypes and 19 (17.1%) patients with MBL-sufficient genotypes (χ^2^, p = 0.67). Regarding the *MASP2* genotype, post-stroke infections appeared in 2 (13.3%) patients with D105>G SNP and in 22 (18.3%) patients with the wild-type genotype (χ^2^, p = 0.63). The risk of infection after stroke was not associated with the serum levels of MBL at day 0 (p = 0.47) or at day 90 (p = 0.95), the levels of MASP-2 at day 0 (p = 0.07) or at day 90 (p = 0.14), the MBL genotype (OR = 1.27, 95% CI 0.42–3.84, p = 0.67), or the *MASP2* genotype (OR = 0.68, 95% CI 0.14–3.25, p = 0.63).

## Discussion

The complement is an integral component of the innate immune system that has been implicated in the pathophysiology of acute stroke [3], [4], [33]. In this study, we report for the first time the important contribution of the lectin pathway of complement activation to brain tissue fate after acute stroke, both in mice and humans. All the patients included in this study participated in a randomized, double-blind, controlled trial that limited the likelihood of clinical bias, and the main clinical findings of the study were in consonance with the results in experiments conducted in WT and MBL-null mice.

MBL-null mice disclosed after stroke smaller infarctions, and better functional outcome than WT mice. This effect was associated with attenuation of complement deposition and neutrophil accumulation into the ischemic brain tissue in MBL-null versus WT mice. The contribution of MBL to ischemic brain damage was further confirmed when MBL was exogenously reconstituted in MBL-null mice. In agreement with these findings in experimental animals, the main clinical contribution of this study was that *MBL2* genotypes, which were associated with low circulating levels of functional MBL protein oligomers, increased approximately 11 times the odds of good functional outcome after stroke. Indeed, carriers of this genetic trait doubled the chance of becoming functionally independent stroke survivors, independently of the effects of established key prognostic factors such as age, gender, and initial stroke severity. Importantly, the benefits of a MBL-low genotype on stroke recovery were not counterbalanced by an increased risk of infections which have been recently associated with a stroke-induced immune-depression syndrome [34]. These results concur with a recent study suggesting that MBL is involved in the protective effect of C1 inhibitor in a murine model of brain ischemia/reperfusion [15].

Unlike the major contribution of MBL, the study did not unravel a significant involvement of *MASP2* genotype or MASP-2 levels in the prognosis of stroke. It has been speculated that MASP-2 deficiency might have broader consequences than MBL deficiency because MASP-2 is involved in the biological activity of not only MBL but also ficolins, a group of functionally related proteins [32]. However, we did not find an association between heterozygous *MASP2* variant genotypes and stroke outcome. Since heterozygosity for the *MASP2* variant allotypes does not lead to significant reduction in the function of the lectin pathway, it is likely that the D105>G variant is only relevant in homozygousity, which, with a gene frequency of about 5%, is a rare condition [20]. Nonetheless, on the basis of our findings we cannot fully exclude the possibility that MBL might have effects independent of MASP-2, as suggested by several findings. For instance, MBL stimulates macrophage phagocytosis in a manner independent of MASP-2 [35]. This matter deserves further investigation.

The inflammatory and lytic effects of complement may significantly contribute to tissue damage and MBL may modulate inflammation and trigger pro-inflammatory cytokine release from monocytes [36]. Although we found no significant associations between the *MBL2* genotype and the total number of circulating monocytes or other white blood cells, we did not assess the phenotype of monocytes in this cohort. However, recent studies by our group have shown assorted effects of stroke on circulating monocytes including an early rise of minor subpopulations with less inflammatory drive and increased capacity for tissue repair [37]. Therefore, further investigation will be required to identify the phenotypic traits of monocytes of subjects with MBL-low genotypes.

The study identified that the individuals carrying MBL-low genotypes had lower peaks of CRP, C3 and C4 in serum, and a strong correlation between CRP, C3 and C4 complement proteins, in agreement with experiments indicating that the complement-activating functions of CRP and MBL are coordinated [38]. CRP is a classic acute phase reactant that belongs to the pentraxin family of calcium dependent ligand-binding plasma proteins that play a role in the humoral innate response. CRP binds with high affinity to phosphocholine residues and with a variety of autologous and extrinsic ligands which are more extensively exposed in or on damaged cells and tissues (reviewed in [39]). Animal studies have shown that CRP can enhance ischemic tissue damage by a complement-dependent mechanism in the heart [40] and brain [41]. The results of our study indicate that the deleterious effects of MBL after acute stroke are associated with increased concentrations of CRP, although additional studies will be required to determine whether there is a causal effect.

Patients with MBL-low genotype also disclosed a cytokine profile in blood characterized by a predominance of anti-inflammatory over pro-inflammatory cytokines. Furthermore, the persistence of this anti-inflammatory profile at 3 months after stroke was in stronger support of a genetic trait rather than an acute-phase reaction. It can be argued that the improved functional recovery and increased brain tissue survival associated with a MBL-low genotype were related in part with a cytokine environment that allowed brain cells to be exposed to stronger anti-inflammatory signaling [42].

In summary, this study shows the major relevance of the MBL pathway of complement activation in acute ischemic or hemorrhagic stroke. Although the mechanisms that lead to ischemic and hemorrhagic stroke may differ, our results are in agreement with the concept of a danger model in which signals of cell injury sensed by immune receptors trigger shared immune responses [43]. Our data indicate that a genetically-defined MBL deficiency facilitates anti-inflammatory responses after acute stroke that result in long lasting beneficial effects on post-stroke functional recovery. Validation of these results would justify the quest of therapeutic interventions aimed to specifically inhibit the lectin pathway of complement activation in patients with acute stroke.

## Supporting Information

Text S1 Extended methods
(0.04 MB DOC)

Figure S1 Complement system activation in carriers of D105>G and WT MASP2-genotypes. Serum concentration (g/L) of C3 and C4 at day 0 (d0) (n = 96) and day 2 (d2) (n = 96). Values are represented as meanÂ±SD.
(2.05 MB TIF)

Figure S2 Balance between T helper (h) 1 cytokines and Th2 cytokines and clinical outcome. Serum measurements at day 0 (d0) (n = 129); day 1 (d1) (n = 121); day 2 (d2) (n = 122); day 3 (d3) (n = 116); day 4 (d4) (n = 111), day 7 (d7) (n = 101), and day 90 (d90) (n = 92). Values are mean Â± SD. *p<0.05.
(0.67 MB TIF)

Table S1 Main traits in patients with ischemic or hemorrhagic stroke.
(0.03 MB DOC)
