# Supplementary material for: Genetically-Defined Deficiency of Mannose-Binding Lectin Is Associated with Protection after Experimental Stroke in Mice and Outcome in Human Stroke
Source: PLoS One. 2010 Feb 3;5(2):e8433. doi: 10.1371/journal.pone.0008433 (PMC2815773; doi:10.1371/journal.pone.0008433)
Supplement: Text S1 — Extended methods (0.04 MB DOC) [file pone.0008433.s001.doc]

**Supplementary Information**

**Extended Methods**

**Animals and brain ischemia**

Four-month-old male MBL-null mice (B6.129S4-Mbl1tm1Kata Mbl2tm1Kata/J) were obtained from The Jackson’s Laboratory. MBL-A mutant homozygotes were bred with homozygous MBL-C mutants to generate mice homozygous for both mutations (MBL-null). Double mutant mice were backcrossed to C57BL/6J for 7 generations before being made homozygous. Age-matched male C57BL/6J wild-type (WT) mice were used as controls. Animal work was approved by the local Ethical Committee (CEEA, University of Barcelona) and performed in compliance with the Spanish and European legislations. Mice were anesthetized with isoflurane by the aid of a facial mask. Brain ischemia was induced by two-hour transient occlusion of the middle cerebral artery using an intraluminal technique and followed by reperfusion, as previously reported [22]. Body temperature was monitored during surgery and maintained at 37 ºC. Following reperfusion, mice were allowed to recover and kept for 48h.

A neurological test was carried out at 48h to assess the neurological function in the mice. Five categories were tested by evaluating body symmetry, front limb symmetry, gait, compulsory circling, and circling behaviour. Each category was assigned a score from 0 (not affected) to 4 (the most affected). The final score was the sum of all categories. Infarct volume was evaluated [22] at 48 h by a researcher that was blind to the animal groups. After this test, animals were anesthetized and killed. The brain was removed and cut in 1mm-thick coronal sections with a tissue-slicer. Brain slices were immersed in a 1% solution of 2,3,5-triphenyltetrazolium chloride (TTC) for 10 min at 37ºC. Sections were then immersed overnight in a 4% paraformaldehyde solution in phosphate buffer and washed in phosphate buffer. Ischemic tissue gave no reaction with the mitochondrial TTC staining, which turns strong red in healthy tissue while keeping infarcted tissue pale or white. Brain sections were scanned and given a code that did not reveal the type of animal. Then, the area of infarction was measured by a researcher that was blinded to the animal groups. Infarction was determined by measuring the white or pale area in each section using Scion Image software (based on NIH Image). A correction for oedema was made by multiplying the infarct area by the ratio of the contralateral to the ipsilateral hemisphere. Infarct volume was calculated by integrating the areas of all sections of each animal. In each brain section, we calculated the percentage of hemispheric area that was infarcted by dividing the infarct area by the area of the contralateral hemisphere *100.

**Immunohistochemistry and Western blotting in mouse brain tissue**

At 48h postischemia, mice were deeply anesthetized and killed. The brain was removed and immersed in isopentane at -40ºC. 14 μm-thick frozen brain sections were cut in a cryostat. Immunohistochemistry was performed with a rabbit polyclonal antibody against C3 (#Ab11887, Abcam) that was used diluted 1:20. Sections were counterstained with Hoechst to visualize the nuclei of the cells. For quantification of C3 immunoreaction, the percent immunopositive area for each animal was measured using AnalySIS Software (Soft Imaging System). Quantification of C3 deposition was made in sections taken at three different coronal sections form Bregma +2mm to Bregma -3 mm. Images were taken from 3 fields and the mean value was calculated for each animal. The area of each field was 0.32 mm^2^. Images were taken from the zones showing the highest intensity of fluorescence per each section. The sections were labeled with a code that did not reveal the identity of the animals. Images were obtained with the x40 objective. Fluorescence images were transformed to black and white images (from 0 to 256 grey levels) that were segmented into immunopositive and immunonegative zones according to a fixed threshold that was set at grey level 40. The value for each animal was the mean of three values obtained from areas of the ischemic region. To visualize the location of C3 in relation to blood vessels, immunoreactions were carried out with a rabbit polyclonal antibody against laminin (1:400) (DakoCytomation, Denmark) that stains the basal lamina, and against CD11b (1:100) (Serotec). Secondary antibodies were from Invitrogene (Molecular Probes, Alexa Fluor 546, 488, and 680, for red, green and blue fluorescence, respectively). Fluorescence was observed under a confocal microscope (Leica DM5500Q, Leica Microsystems CMS GmbH, Wetzlar, Germany).

Brain protein extracts were obtained and processed for Western blotting as previously reported [22]. Mice were anaesthetized and subjected to euthanasia, the brain was removed and the ischemic and contralateral cortex and striatum were dissected out and rapidly frozen and kept at –80 ºC. Tissue samples were homogenized in radioimmunoprecipitation assay buffer (RIPA), containing 0.01 M phosphate-buffered saline (PBS), 0.1% sodium dodecyl sulphate, 0.5% sodium deoxycholate, 1% non-ionic detergent Igepal, and a cocktail of protease inhibitors (Complete, Boehringer Mannheim, Germany), according to instructions of the manufacturer. All products and reagents, unless otherwise stated, were from Sigma. Samples were kept on ice for 30 minutes and then centrifuged at 12,000 X g at 4ºC for 15 min, and the supernatants were used as the total protein fraction. The protein concentration was determined with the Bradford assay (Bio-Rad, Hercules, CA, USA). Thirty µg of the protein extracts were denatured at 100ºC for 5 min in the presence of β-mercaptoethanol, and then loaded in 10% polyacrylamide gels. Proteins were transferred to a polyvinylidene difluoride membrane (Immobilon-P, Millipore, Bedford, MA, USA), which was incubated overnight at 4ºC with rabbit anti-mouse antibodies against C3: one of them was purchased from Abcam (#Ab11887) and was used diluted 1:500, while the other was from Santa Cruz Biotechnology Inc. (Temecula, CA) (#H-300, sc-20137) and was used diluted 1:1000. A mouse monoclonal antibody against myeloperoxidase (MPO) (Pharmacia) was used diluted 1:500. On the following day membranes were incubated for 1 h with an anti-mouse Ig peroxidase-linked secondary antibody (1:2000) (Amersham). The reaction was visualized using a chemiluminescence detection system based on the luminol reaction. Mouse mAb against either glyceraldehyde 3-phosphate dehydrogenase (GAPDH) diluted 1:5,000 (Assay designs, Ann Arbor, MI, USA) or β-Tubulin (Sigma) diluted 1:100,000 were used as loading controls. The intensity of the bands was measured by densitometric analysis (GS-800 Densitometer, Bio-Rad). The ratio between C3 band intensity to the band intensity of the corresponding loading control was calculated to correct for any variation in protein gel loading. Values in the ischemic groups are expressed as percentage of non affected control.

**Sequencing genomic DNA**

At day 0, genomic DNA was extracted from ethylenediaminetetraacetic (EDTA)-treated whole blood human samples using the QIAmp DNA blood mini kit following manufacturer’s instructions (QIAGEN GmbH, Hilden, Germany) and then stored at -20ºC until analysis. *MBL2 and MASP2* genotyping was performed using a previously reported sequence-based typing technique [27]. Sequencing reactions were analyzed by capillary electrophoresis in an ABI Prism 3100 Genetic Analyzer (Applied Biosystems, Warrington, UK). SNPs at codons 52, 54, and 57 (named D, B, and C variants, respectively) of exon 1 of *MBL2* gene are major determinants of serum MBL levels [17, 19]. These structural variants are collectively named 0, whereas A indicates the WT variant. Three additional SNPs at positions -551 (H/L), -221 (X/Y) and +4 (P/Q) in the 5’-flanking region of *MBL2* gene also influence serum MBL levels in individuals with the WT variant [19]. According to previous studies [19, 28, 29], genotypes 0/0, 0/XA, and XA/XA were classified as MBL-low variants while the remainder were MBL-sufficient variants.

**Serum levels of MBL and MASP-2**

Human serum was diluted 100-fold and applied onto microtitre wells pre-coated with mannan from baker’s yeast. Bound MBL was detected with biotin-labeled monoclonal antibody (mAb) against MBL, followed by europium-labeled streptavidin and TRIFMA. Detection limit was 10 ng MBL/ml [30]. For determination of MASP-2, serum was diluted 40-fold and applied onto wells coated with 8B5 mAb directed against MASP-2 C-terminal domain. Bound MASP-2 was detected with biotin-labeled 6G12 mAb against the N-terminal domain of MASP-2, followed by europium-labelled streptavidin and TRIFMA measurement [31].

## Serial changes of inflammatory parameters in blood samples of stroke patients

Human blood samples were collected at baseline (day 0) and days 1, 2, 3, 4, 7, and 90, and stored at -80ºC until analysis by investigators blinded to clinical and radiological data. Parameters assessed in the participants included neutrophil, lymphocyte and monocyte counts (Pentra DX 120; ABX Diagnostics), C-Reactive Protein (CRP) (Dade-Behring, Newark, NJ), tumor necrosis factor (TNF)-α, interleukin (IL)-10 and IL-6, with commercially available quantitative sandwich enzyme-linked immunoadsorbent assays (Quantikine, R&D System, Minneapolis, MN). C3 and C4 complement proteins were measured by nephelometry (BN ProSpec System, Dade Behring, Marburg, Germany) at day 0 and day 2 (n=96 per time point).
