# Supplementary figures and images for: Genetically-Defined Deficiency of Mannose-Binding Lectin Is Associated with Protection after Experimental Stroke in Mice and Outcome in Human Stroke
Source: PLoS One. 2010 Feb 3;5(2):e8433. doi: 10.1371/journal.pone.0008433 (PMC2815773; doi:10.1371/journal.pone.0008433)

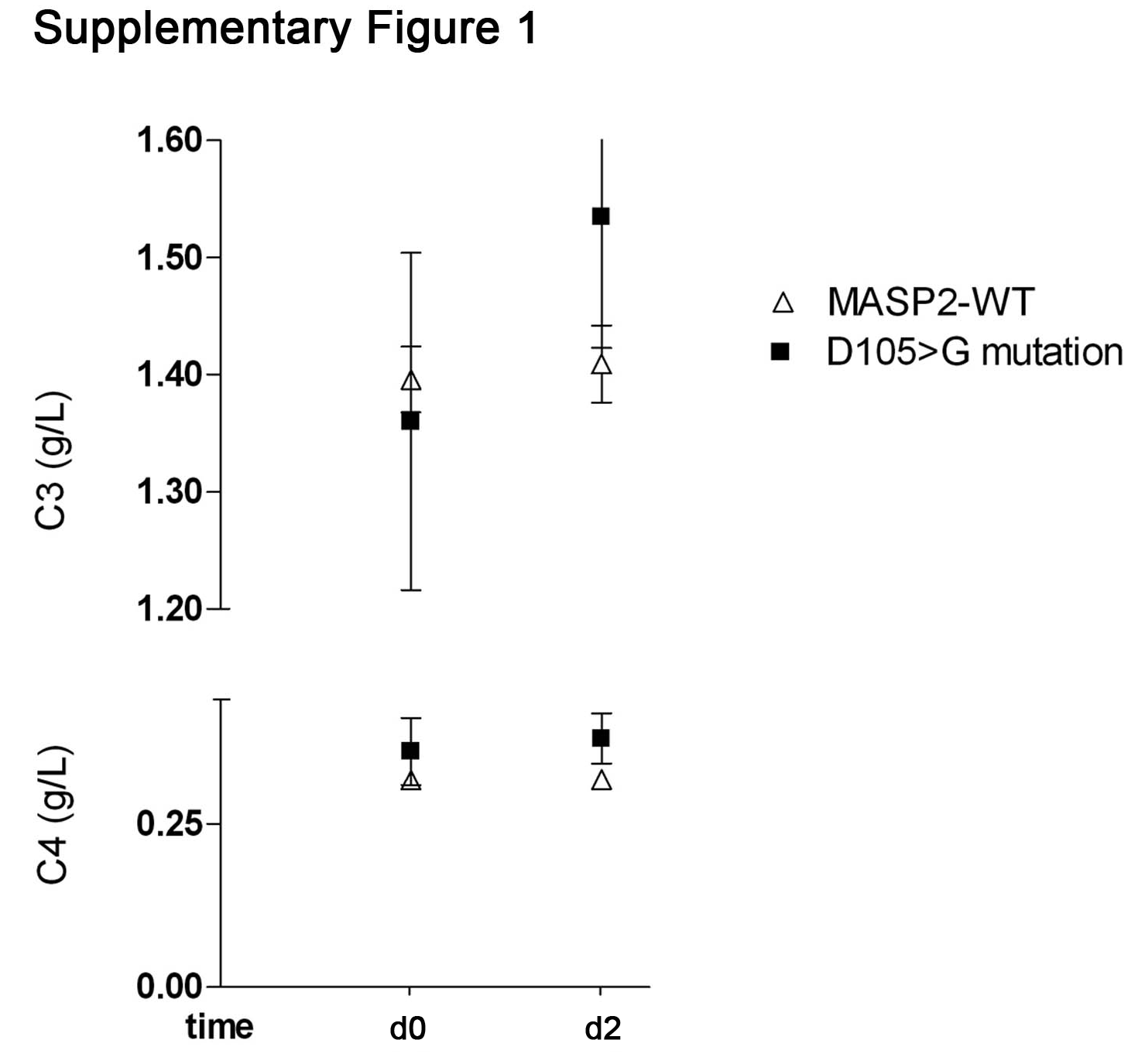

Supplement: Figure S1 — Complement system activation in carriers of D105>G and WT MASP2-genotypes. Serum concentration (g/L) of C3 and C4 at day 0 (d0) (n = 96) and day 2 (d2) (n = 96). Values are represented as meanÂ±SD. (2.05 MB TIF) [file pone.0008433.s002.tif]

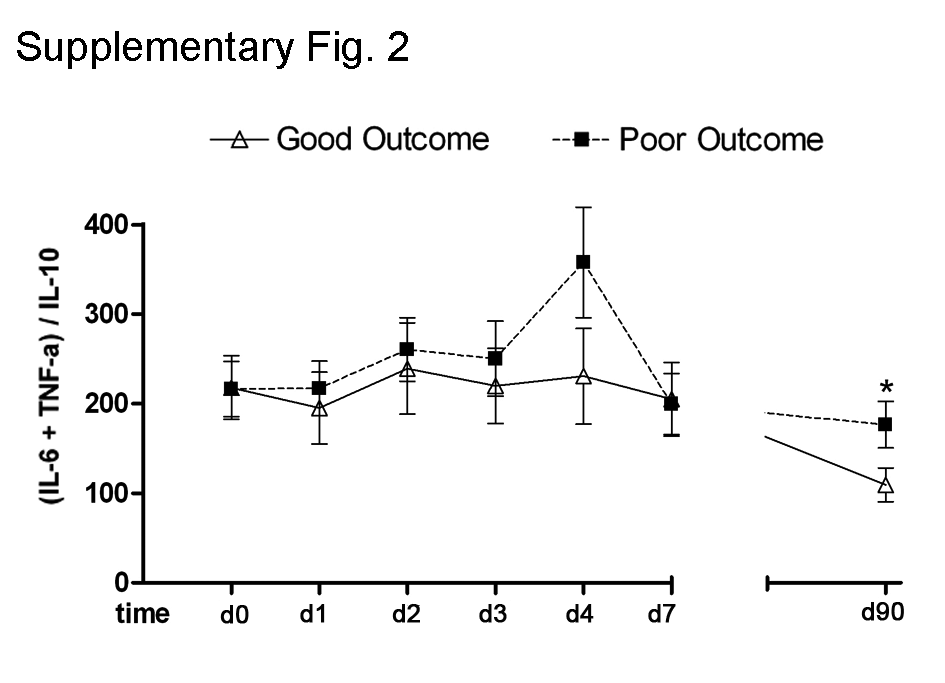

Supplement: Figure S2 — Balance between T helper (h) 1 cytokines and Th2 cytokines and clinical outcome. Serum measurements at day 0 (d0) (n = 129); day 1 (d1) (n = 121); day 2 (d2) (n = 122); day 3 (d3) (n = 116); day 4 (d4) (n = 111), day 7 (d7) (n = 101), and day 90 (d90) (n = 92). Values are mean Â± SD. *p<0.05. (0.67 MB TIF) [file pone.0008433.s003.tif]
