# Supplementary material for: Genetically-Defined Deficiency of Mannose-Binding Lectin Is Associated with Protection after Experimental Stroke in Mice and Outcome in Human Stroke
Source: PLoS One. 2010 Feb 3;5(2):e8433. doi: 10.1371/journal.pone.0008433 (PMC2815773; doi:10.1371/journal.pone.0008433)
Supplement: Table S1 — Main traits in patients with ischemic or hemorrhagic stroke. (0.03 MB DOC) [file pone.0008433.s004.doc]

**1. Supplemental TableI: Main traits in patients with ischemic or hemorrhagic stroke**

**Ischemic stroke Hemorrhagic stroke p value**

N=109 (81%) N=26 (19%)

**Demographics, risk factors**

Age (mean, SD), yrs 73.6 (11.9) 70.5 (10.5) 0.22

Male, n (%) 52 (48) 16 (62) 0.27

Active smoking, n (%) 16 (15) 7 (27) 0.31

Hypertension, n (%) 73 (67) 12 (46) 0.05

Diabetes, n (%) 23 (21) 7 (27) 0.60

Coronary heart disease, n (%) 16 (15) 1 (4) 0.19

Previous stroke, n (%) 20 (18) 3 (12) 0.56

Peripheral artery disease, n (%) 11(10) 0 (0) 0.12

**Baseline data**

NIHSS score, mean (SD) 13.6 (6.7) 13.4 (6.4) 0.69

Systolic BP (mean, SD), mm Hg 160.5 (30.7) 175.1 (34.8) 0.03

Diastolic BP (mean, SD), mm Hg 87.7 (18.9) 91.0 (24.2) 0.44

Glucose (mean, SD), mg/dL 143.1 (53.2) 147.3 (39.3) 0.70

**Clinical course**

Infection day 7, n (%) 17 (16) 7 (42) 0.25

Infection day 90, n. (%) 32 (29) 11 (42) 0.24

Death, day 90, n (%) 19 (17) 6 (23) 0.57

Favorable outcome day 90, n (%) 36 (33) 11 (42) 0.37

**Complement proteins**, g/L (mean, SD)

C3 at baseline 1.41 (0.30) 1.34 (0.53) 0.38

C3 at 48 h 1.39 (0.28) 1.37 (0.26) 0.49

C4 at baseline 0.33 (0.13) 0.31 (0.10) 0.59

C4 at 48 h 0.33 (0.11) 0.31 (0.10) 0.39

**C-reactive protein, mg/dl mean (SD)**

Baseline 1.14 (2.85) 0.67 (0.78) 0.42

day 1 1.71 (3.09) 1.94 (2.63) 0.74

day 2 2.50 (3.55) 4.14 (8.89) 0.14

day 3 2.77 (3.43) 3.83 (5.61) 0.23

day 4 2.90 (3.43) 3.30 (6.35) 0.68

day 7 2.79 (3.69) 2.63 (5.79) 0.88

day 90 0.65 (1.06) 0.91 (1.27) 0.42

mbl-low, n (%) 18 (16.5) 6 (23.0) 0.30

a105g-masp2, n (%) 11 (11.0) 3 (11.5) 1.00

**mbl, ng/ml mean (SD)**

Baseline 1304,88 (1255,18) 1535,34 (1275,68) 0.47

day 90 1636,68 (1397,64) 2429,68 (1907,72) 0.16

**masp2, ng/ml mean (SD)**

Baseline 357,91 (187,62) 429,70 (269,51) 0.54

day 90 369,04 (175,60) 469,08 (248,10) 0.12
